# Environmental Health Impacts of CAFOs

**DOI:** 10.1289/ehp.10124

**Published:** 2007-07

**Authors:** Liz Wagstrom

**Affiliations:** National Pork Board, Clive, Iowa, E-mail: Lwagstrom@pork.org

I am concerned about the development and content of the Mini-Monograph on Environmental Health Impacts of Concentrated Animal Feeding Operations (CAFOs) (Bunton et al. 2007; Burkholder et al. 2007; Donham et al. 2007; Gilchrist et al. 2007; Heederik et al. 2007; Thorne et al. 2007). Although I believe that the process of developing these reports was flawed, I will limit my comments to the content of the reports. It is difficult to comment on six separate articles within the word limitations of a letter; therefore, I will make some general comments followed by a few specific examples.

Overall, there was a wide variation between the six articles (Bunton et al. 2007; Burkholder et al. 2007; Donham et al. 2007; Gilchrist et al. 2007; Heederik et al. 2007; Thorne et al. 2007) in both tone and scientific rigor. However, generally the authors did not differentiate between potential risks associated with general animal production—regardless of facility type—and risks associated with CAFOs. Their default was to assign any potential risk to the attribute “CAFO” rather than the attribute “animal.” For example, Burkholder et al. (2007) gave the impression that pathogens in manure would not exist in manure from animals raised in facilities that do not meet the definition of a CAFO. The authors of these articles generally referred to risk as the presence of potential pathogens or toxic substances. A true assessment of potential risk requires an assessment of exposure as well as volume or presence (National Research Council 1994), something that was not addressed to any extent in these reports.

Additionally, Burkholder et al. (2007) did not in any way differentiate between the normal operation of a CAFO and potential impacts on water quality versus the results of a single catastrophic event, such as failure of a lagoon wall. Repeated reference to a single catastrophic event involving a single lagoon does not aid scientists or the public in understanding how CAFOs are operated on a daily basis, or how the typical handling of manure under daily operation may impact water quality. The authors did not attempt to document regulatory oversight or best management practices that have been implemented to minimize potential negative impacts on the environment (U.S. Environmental Protection Agency 2002). Indeed, there was no mention of the relative rarity of lagoon failure, leaving the impression that this is a common event.

In some cases, where the authors attempted to assign risk to CAFOs rather than to animals, they presented biased opinion rather than fact. For example, Gilchrist et al. (2007) stated that

Pathogens tend to be amplified in animals raised in CAFOs and, thus, are more difficult to eliminate in meat packing processes.

This blanket statement does not specify a type of pathogen, but studies indicate that—among the more important pathogens—this is an inaccurate statement. For example, pathogens such as *Trichinella spiralis*, formerly one of the most prominent pork-associated pathogens, have largely disappeared with the movement of pigs to indoor production (Roy 2003). Furthermore, pork carcass contamination with bacterial pathogens such as *Salmonella* is consistently lowest in the large packing plants, which because of the large volume of production are most likely to acquire animals from large producers (U.S. Department of Agriculture, Food Safety and Inspection Service 2006). This clearly invalidates the argument that these pathogens are more difficult to eliminate in the packing process.

Gilchrist et al. (2007) also referred to Denmark as a country that has experienced a successful transition to antibiotic-free production. This is incorrect, and indeed the latest DANMAP report (Danish Integrated Antimicrobial Resistance Monitoring and Research Program 2006) indicates that therapeutic antibiotic use in agriculture now exceeds the amount of antibiotics that were used to promote feed conversion before a ban on antibiotic growth promotion. Additionally, pig mortality in Denmark has risen 25% in the last 10 years (Agence France-Presse 2005).

Gilchrist et al. (2007) suggest that industry leaders should take a leadership role in antibiotic use, but they apparently were unaware or intentionally overlooked the fact that the American Veterinary Medical Association, the American Association of Swine Veterinarians (2004), and the National Pork Board (2000) have had prudent use guidelines for 7 years, and that in 2005 the pork industry launched the Take Care — Use Antibiotics Responsibly program (National Pork Board 2005). More than 50 million pigs are marketed each year by producers who recognize the importance of protecting public health and animal health and welfare through the responsible use of antibiotics by pledging their support for the Take Care program.

The topics covered by *EHP* are important and worthy of public discussion and scientific investigation. However, we do the public, producers, and the research community a disservice when that discussion is driven by misinformation and subjective opinion.
